# Exploring the Implications of New-Onset Diabetes in COVID-19: A Narrative Review

**DOI:** 10.7759/cureus.33319

**Published:** 2023-01-03

**Authors:** Joseph Pergolizzi, Jo Ann K LeQuang, Frank Breve, Peter M Magnusson, Giustino Varrassi

**Affiliations:** 1 Cardiology, Native Cardio, Inc., Naples, USA; 2 Pain Management, NEMA Research, Inc., Naples, USA; 3 Pharmacy, Temple University, Philadelphia, USA; 4 Cardiology, Institution of Medical Sciences, Örebro University, Örebro, SWE; 5 Institute of Medicine, Karolinska Institutet, Stockholm, SWE; 6 Research, Paolo Procacci Foundation, Roma, ITA

**Keywords:** diabetes mellitus, covid-associated diabetes, type 3 diabetes, diabetes, covid-19

## Abstract

Post-viral new-onset diabetes has been an important feature of the COVID-19 pandemic. It is not always clear if new-onset diabetes is the unmasking of a previously undiagnosed condition, the acceleration of prediabetes, or new-onset diabetes that would not have otherwise occurred. Even asymptomatic cases of COVID-19 have been associated with new-onset diabetes. Diabetes that emerges during acute COVID-19 infection tends to have an atypical presentation, characterized by hyperglycemia and potentially life-threatening diabetic ketoacidosis. It is not always clear if new-onset diabetes is type 1 or type 2 diabetes mellitus. Many cases of COVID-associated diabetes appear to be type 1 diabetes, which is actually an autoimmune disorder. The clinical course varies temporally and with respect to outcomes; in some cases, diabetes resolves completely or improves incrementally after recovery from COVID-19. Disruptions in macrophagy caused by COVID-19 infection along with an exaggerated inflammatory response that can occur in COVID-19 also play a role. Those who survive COVID-19 remain at a 40% elevated risk for diabetes in the first year, even if their case of COVID-19 was not particularly severe. A subsequent post-pandemic wave of new diabetes patients may be expected.

## Introduction and background

During the prior coronavirus epidemics of SARS-CoV-1 (SARS) in 2003 and the Middle-East Respiratory Syndrome (MERS) in 2012, diabetes emerged as a recognized risk factor for poor outcomes [1]. In these two epidemics, pre-existing type 2 diabetes mellitus (T2DM) in particular was recognized as a contributor to disease severity and lethality [2-4]. Perplexing cases of new-onset diabetes, sometimes with atypical presentations, occurred in SARS patients with no history of diabetes, defying ready explanations [2]. This has occurred again in COVID-19 but to a larger degree given the millions affected by the pandemic. Inflammatory diseases, including but not limited to COVID-19, may be associated with transient hyperglycemia. There are three plausible explanations, none of which precludes the others. The first is that hyperglycemic and diabetes-mimicking events in COVID-19 patients are transient and the expected consequences of hyperinflammation. The second is that the inflammatory and possibly other symptoms of COVID-19 unmask and possibly accelerate undiagnosed diabetes. Finally, there is a plausible notion that new-onset diabetes in COVID-19 patients is a new form of trauma-induced diabetes [5].

Studying new-onset diabetes during a global pandemic involves numerous challenges. Pre-existing diabetes and obesity, a risk factor for diabetes, are common comorbidities associated with COVID-19. Many COVID-19 patients may have well been undiagnosed diabetics, prediabetics, or disposed to diabetes [6,7]. However, observations that the SARS-CoV-2 virus can enter the islet cells of the pancreas and damage β-cells, triggering hyperglycemic episodes, has led to a discussion of “new-onset diabetes,” sometimes erroneously called “type 3 diabetes,” which mimics type 1 diabetes mellitus (T1DM) and may be reversible [8]. As the pandemic weakens its grip, it is important to sort through the sometimes conflicting information regarding how COVID-19 might be connected to diabetes [9] and the emergence of what could be a new form of diabetes relevant to inflammatory diseases. This narrative review surveys what is currently known and not known about what might be called new-onset diabetes in the context of COVID-19.

## Review

Methods

The PubMed and Google Scholar databases were searched using keyword phrases, “new onset diabetes COVID,” “type 3 diabetes COVID,” “COVID type 2 diabetes,” and “COVID type 1 diabetes.” The bibliographies of particularly relevant articles were reviewed. Using the PubMed database feature of “related articles,” relevant articles on similar or related topics were reviewed. The Cochrane Library database was searched separately although many results overlapped with previous findings. Altogether, there were 320 results that addressed the relationship between COVID-19 and new-onset diabetes, of which 80 were used in the narrative review. Inclusion criteria were that the article be in English, be a peer-reviewed article, and address COVID-related diabetes; randomized clinical trials, clinical trials, reviews, and meta-analyses were included but correspondence, editorials, case reports, and articles not in English were excluded. For the two tables of case studies, the PubMed database was again searched for “COVID-19, case studies, new-onset diabetes” and 17 relevant results were returned. Case reports likewise had to be in English and published in a peer-reviewed journal, relating to new-onset diabetes in the context of COVID-19. Authoritative websites, such as the site of the Centers for Disease Control and Prevention, were also consulted for relevant statistical data.

Results

An international registry of patients with new-onset diabetes following acute COVID-19 infection, COVIDIAB, has been established to better explore the complex and bidirectional relationship between COVID-19 and diabetes [10]. In patients without a history of T2DM and not considered to be prediabetic, metabolic decompensation in COVID-19 infection can initiate T1DM [11]. Unlike T2DM, T1DM is an autoimmune disease, in which auto-reactive CD4+ and CD8+ T cells destroy the β-cells of the pancreas so that insulin is not secreted. While genetics was long thought to play a major role in T1DM, environmental influences may be far more influential than previously understood [12]. For example, immigrants tend to have rates of T1DM that reflect their destinations rather than their ethnic origins, suggesting that environmental factors may override genetic ones [12].

Viral infections have the potential to both damage or protect β-cells, and this is not limited to COVID-19 although it has emerged most prominently during the pandemic [8]. Viral replication could lead to lysis that damages the β-cells but, the body’s inflammatory response might likewise drive auto-immunity and auto-reactive CD+ T cells [8].

Fulminant T1DM, a new and rare subtype of T1DM, occurs when a very large amount of β-cells are destroyed rapidly [13]. Fulminant T1DM has been reported to be associated with other types of viral infections, such as enterovirus Coxsackie infection, hepatitis A, influenza B, and others [14-16]. Fulminant T1DM has also been reported following COVID-19 vaccination [17]. It is not clear if new-onset diabetes in the context of COVID-19 is fulminant T1DM or another type, but certainly, it is plausible that some of this new-onset COVID-related diabetes is fulminant T1DM. In any viral-induced cases of new-onset fulminant T1DM, there is a paroxysmal onset of hyperglycemic ketoacidosis, a short course of classic diabetes symptoms (polyuria, polydipsia, unintentional weight loss), a lack of islet-related auto-antibodies, low C-peptide levels, and high serum pancreatic enzyme levels [8]. This viral-induced fulminant T1DM has exhibited the potential to resolve completely, providing there is no extensive damage to β-cells in the pancreas, or to improve substantially with time [8]. The reasons behind chronic β-cell destruction are unclear, but it has been suggested that the viral epitope mimics the host islet protein, inducing cross-reactivity and an autoimmune T-cell response that fights the host [18]. While this theory may explain the role of the virus in β-cell damage, it does not explain how the virus initiates a pancreatic invasion [18]. Cytokine storm may be a possible explanation. The release of pro-inflammatory cytokines may accelerate β-cell destruction, impair glucose-mediated insulin release, and impede the conversion of proinsulin into insulin [8]. This would lead to a diabetes-like disorder.

The limitations and conditions under which β-cells can regenerate after a toxic assault are currently under scientific investigation [19]. The degree and manner in which β-cells are destroyed may play a role, in that the loss of β-cells in diabetes may be gradual and progressive or vast and abrupt [20]. Macrophagy appears to play a critical role in β-cell repair following injury [21].

Hyperglycemia

While hyperglycemia in general is associated with diabetes, episodes of hyperglycemia may occur without diabetes as a result of infection or as a side effect of drugs such as glucocorticosteroids [22]. Hyperglycemia frequently occurs in critical illness, [23] which is defined by a neuro-endocrine response, activity in the sympathetic nervous system, hyperglucagonemia, and the release of cortisol and growth hormone [24]. Individually and in combination, these can trigger insulin resistance even in those without diabetes, leading to hyperglycemia [9]. By the same token, hyperglycemia in response to critical infection may lead to diabetes. In a retrospective review of critically ill patients prior to the pandemic, 28% of those with no history of diabetes but who had severe hyperglycemia in the hospital developed new-onset diabetes, while only 4% with normal-range glucose in the hospital developed new-onset diabetes [23].

The American Diabetes Association defines new-onset hyperglycemia without diabetes as a fasting plasma glucose level between 5.6 and 6.9 mmol/L (100 to 125 ml/dL) or an HbA1c score between 5.7% and 6.4% in the absence of hypoglycemia, or both [25]. Plasma glucose increases when viral stress promotes glucagon production by the ɑ-cells of the islets of Langerhans, increasing hepatic production of glucose. Elevated levels of glucagon adversely affect glucose homeostasis; however, viral stress is unlikely to impact HbA1c levels [25].

Hyperglycemia feeds inflammation, advances coagulation, and disrupts the normal production of various inflammatory mediators [26]. Hospitalized COVID-19 patients with hyperglycemia have higher levels of interleukin-6 (IL-6) and D-dimers [27]. Managing hyperglycemia or at least reducing extreme hyperglycemia can be crucial for the recovery of hospitalized COVID-19 patients [9].

The causes of hyperglycemia in COVID-19 patients likely involve microvascular function. COVID-19 patients may have microthrombi that impede proper glucose disposal [9,28]. The close connection between microvascular function and glycemic control is bidirectional and operates as a feedback loop [28]. Microvascular dysfunction, which can be driven by obesity and other comorbidities, precedes hyperglycemia in T2DM [28]. Microangiopathy can occur in diabetes and has been recognized as a risk factor for worse outcomes with COVID-19 [9]. Microvascular dysfunction plays a role in the development and progression of numerous cardiac, metabolic, and renal diseases and often precedes macrovascular dysfunction, although micro- and macrovascular dysfunction may also occur concurrently [29].

Macrophages serve on the front lines against infection as the body’s key agents of innate immunity [30]. Macrophagy, closely tied to the inflammatory process, includes both M1 and M2 categories of macrophages which are crucial to the inflammatory environment [31]. See Table 1. The transformation of macrophages into M1 or M2 categories is a dynamic process that is not well elucidated but is the subject of current investigation [32]. The body creates M1 or M2 macrophages as needed.

**Table 1:** The two main categories of macrophages. IL, interleukin; Th, T helper; TGF, transforming growth factor; TNF, tumor necrosis factor

Macrophage Category	Inflammatory?	Cytokines	Polarized By
M1	Pro	IL-1β, IL-6, Il-12, IL-23, TNFɑ	Lipopolysaccharide and perhaps in part by Th1 cytokines
M2	Anti	IL-10, TGF-β	Th2 cytokines

Should an infective pathogen invade an organ, M1 phenotypes are released, which send tumor necrosis factor-alpha (TNF-ɑ), interleukin (IL)-1β, IL-12, and IL-23 and other pro-inflammatory cytokines to the site of injury [33]. To prevent organ damage and avoid the risks of prolonged inflammation, the release of M1 cannot be of long duration, so M2 phenotypes then secrete IL-10 and transforming growth factor beta (TGF-β) to brake the inflammatory process and begin homeostasis, remodeling, and tissue repair [33].

Results from an animal study found that the hyperglycemic state polarized macrophages toward the M1 phenotype, leading to prolonged inflammation and excessive production of reactive oxygen species (ROS) [34]. An overabundance of M1 phenotypes can damage tissue and has been associated with abnormal diabetic wound healing [35]. In fact, insulin resistance has been defined at the molecular level as a transition in macrophagy away from the M2 toward M1 [36]. In general, the ratio of M1 to M2 in adipose tissue is considered a marker for the severity of insulin resistance; the more M1, the greater the insulin resistance [37]. Thus, enhancing M1 production at the expense of M2 would likely increase insulin resistance and set the stage for hyperglycemia. Low-grade chronic inflammation in the adipose tissue may be the “missing link” between obesity and T2DM [38]. Moreover, it appears that M1 facilitates the spread of the viral infection in COVID-19, while M2 suppresses it [39]. This would explain why people with diabetes are more vulnerable to COVID-19 infection and have worse outcomes than those without diabetes.

Hyperglycemia in COVID-19 promotes rather than retards inflammation [40]. Excessive inflammatory response worsens outcomes for those with pre-existing diabetes and may set the course for unmasking latent diabetes or inducing new-onset diabetes [40]. Since COVID-19 may adversely affect the complicated interplay of the body’s endocrine, immune, and nervous systems, it allows for an aberrant inflammatory response and potentially dangerous imbalances of serum glucose [41]. In this connection, it is important to consider the “extended autonomic nervous system.” Historically, the autonomic nervous system includes the sympathetic, parasympathetic, and enteric nervous system, but more recently in the wake of COVID-19 the neuro-endocrine and neuro-immune systems have been included to encompass the “extended autonomic nervous system" [42]. The extended autonomic nervous system is a powerful and fast-acting system that can make instantaneous and sometimes extreme life-saving adaptations in the event of serious injury or other conditions, such as hemorrhage. However, the prolonged activation of the extended autonomic nervous system that might occur during acute COVID-19 can lead to prolonged dyshomeostasis and potentially fatal destabilizing effects [42].

Diabetic Ketoacidosis

Diabetic ketoacidosis is a serious and potentially life-threatening condition that occurs when an insulin deficit causes lipolysis and the formation of ketone bodies and acidosis in the blood [43]. Diabetic ketoacidosis is more common in T1DM but may also occur in T2DM, particularly if extreme stress is involved [43,44]. For some COVID-19 patients, diabetic ketoacidosis may be a presenting symptom rather than the conventional COVID-19 symptoms [43]. The exact pathogenesis of diabetic ketoacidosis in COVID-19 patients is not known [43].

Infections, including but not limited to COVID-19, can trigger diabetic ketoacidosis even among those who had no previous history of it [45]. Apart from COVID-19, the annual mortality rate for diabetic ketoacidosis is approximately 5%, which may explain why diabetic patients have higher mortality rates with COVID-19 than patients without diabetes [46]. In a study of 106 pediatric patients with a recent diagnosis of T1DM, 58.5% had diabetic ketoacidosis at baseline; when matched to control patients without T1DM, it was found that the incidence of COVID-19 infection was similar in both groups although the incidence of diabetic ketoacidosis was higher in the T1DM patients [47].

Among COVID-19 patients, diabetic ketoacidosis may occur in those without diabetes [48]. Non-diabetic ketoacidosis is a rare condition that has been observed in starvation, strict dietary regimens, pancreatitis, alcoholism, and lupus [49-51]. Diabetic ketoacidosis is a life-threatening metabolic disruption and associated with hyperglycemia and diabetes, but in COVID-19, diabetic ketoacidosis - sometimes called metabolic ketoacidosis - can occur without diabetes. In a study of 658 hospitalized COVID-19 patients, 6.4% had diabetic ketoacidosis as a presenting symptom. Of this cohort, 27 patients who developed ketoacidosis did not have diabetes [52]. Among those with diabetes, the emergence of diabetic ketoacidosis in COVID-19 suggests a poor prognosis [53].

Diabetic ketoacidosis can create potentially arrhythmogenic electrolyte imbalances, yet there has been a paucity of research into the intersection of COVID-19, diabetic ketoacidosis, and arrhythmic events [54]. Moreover, the incidence of diabetic ketoacidosis increased during the pandemic, but it is not clear if this was a result of COVID-19 infection or the unintended consequences of diverting healthcare resources away from pediatric and diabetic emergencies [55].

Pre-Existing Diabetes

Pre-existing T1DM or T2DM can increase morbidity, extend hospital length of stay, and is associated with higher mortality in patients with viral infections, such as COVID-19 [56-58]. A more specific analysis of this comorbidity is complicated by the fact that T2DM is associated with obesity, hypertension, heart disease, and other conditions known to exacerbate COVID-19 [59]. In a study of COVID-19 patients with previously diagnosed T1DM, over 50% had hyperglycemia, and about a third reported diabetic ketoacidosis [60]. A population-based cohort study found the hazard ratio for COVID-19 mortality was 2.23 for those with pre-existing T1DM and 1.61 for T2DM. These exacerbations were more severe in patients who were over the age of 70, non-Caucasian, had other comorbidities associated with worse outcomes in COVID-19, and who had lower socioeconomic status [61].

Long-standing diabetes may pose less of a risk of COVID-19 infection than newly diagnosed diabetes. Patients with newly diagnosed T2DM who contracted COVID-19 were at a higher risk for all-cause mortality than those with a longer history of diabetes and/or hyperglycemia [22,62]. In a study of 453 COVID-19 patients in China hospitalized in the period from January to March 2020, 11.7% had recently been diagnosed with T2DM, whereas 4.1% had a history of T2DM, and 6.2% had a history of hyperglycemia [62]. Using a multivariable-adjusted hazard ratio for all-cause mortality, the hazard ratios were 3.29 for those with hyperglycemia, 9.42 for those with recently diagnosed T2DM, and 4.63 for those with a history of T2DM [62]. The reasons for these markedly different hazard ratios are not clear.

Unmasking Diabetes

It has been speculated that COVID-19 hospitalization unmasks undiagnosed diabetes, typically T2DM in adults and T1DM in children [63]. Approximately 10% of the U.S. population has T2DM (approximately 37 million people), of whom 20% do not know they have it. The vast majority (80%) of those with prediabetes are unaware of it [64]. T1DM is a much rarer condition, with a global incidence of 15 per 100,000, and has been increasing prior to the pandemic [65,66]. Around the world, about 5% to 10% of all people with diabetes have T1DM [66].

A meta-analysis of seven studies of hospitalized COVID-19 patients found that 19.7% had some form of diabetes [67]. Clearly, some diagnoses of diabetes in COVID-19 patients are the result of the pandemic unmasking undiagnosed diabetes. It is not clear if and how unmasked previously undiagnosed diabetes might differ from new-onset diabetes. However, the presentation of diabetes in COVID-19 is atypical and is characterized by severe insulin resistance, extreme hyperglycemia, and diabetic ketoacidosis [9]. Despite the paucity of research into trying to parse the difference between pre-existing diabetes revealed by COVID-19 versus infection-induced new-onset diabetes, the literature contains numerous case studies. Pediatric patients diagnosed with COVID-19 either as primary or incidental diagnoses were sometimes discovered to have diabetes, typically T1DM. See Table 2.

**Table 2:** Case studies of pediatric patients diagnosed with diabetes in the course of acute COVID-19 infection. All cases in this table were diagnosed with COVID-19 although some patients were asymptomatic. Many of these studies did not specify what type of diabetes was diagnosed; where T1DM or T2DM was stated, it is included in the table. Studies are listed in alphabetic order by last name of the first author [68-79]. yo, years old

Case Study	Patient(s)	Presentation	Case Notes
Aabdi 2021 [68]	3 yo boy	Unresponsive, tachycardia, diabetic ketoacidosis	Full recovery, managed post-discharge on insulin
Badawy 2022 [69]	13 yo girl	Polyuria, polydipsia, weight loss; diabetic ketoacidosis; positive COVID test but asymptomatic	Recovered in five days, T1DM diagnosed, discharged with insulin
Basta 2021 [70]	3 yo boy	Severe hyperglycemia, metabolic acidosis, positive COVID-19 test	Managed with insulin drip, diagnosed new-onset T1DM
Benyakhlef 2021 [71]	3 yo boy	Acute dyspnea, diabetic ketoacidosis	Recovered, new-onset T1DM
Chekhlabi 2021 [72]	4 yo girl	Polydipsia, polyuria, exposed to COVID-19 but with mild symptoms	Developed diabetic ketoacidosis which resolved in 24 hours, full recovery, requires insulin
	7 yo boy	Diabetic ketoacidosis, asymptomatic for COVID-19	Full recovery, required insulin after discharge but dose has been incrementally decreased
Duong-Quy 2022 [73]	13 yo boy	Admitted to COVID-19 ICU with fever, cough, dyspnea, confusion, and hyperglycemia; developed diabetic ketoacidosis in hospital	Diagnosed with new-onset T1DM; discharged after 16 days with insulin; recovered but still has T1DM
Kastner and Harsch 2021 [74]	8 yo boy	Polyuria and asymptomatic COVID-19	New-onset T1DM diagnosed and treated with insulin; COVID-19 antigens were found in blood 15 days later although he never had symptoms
Lanca et al. 2022 [75]	13 yo boy	Persistent hyperglycemia and high insulin requirements; diabetic ketoacidosis; mild COVID-19 symptoms	New-onset T1DM which required insulin post-discharge. His mother has T1DM
	8 yo male	Severe COVID-19 with respiratory symptoms; hyperglycemia	Although hyperglycemia resolved after COVID-19, he needed low doses of insulin after discharge
Naguib 2020 [76]	8 yo girl	COVID-19 diagnosis with multisystem inflammatory syndrome, hyperglycemia, ketosis, metabolic acidosis	Treated with infliximab and recovered from COVID-19, discharged after 10 days, required insulin but dose has been lowered to 1.1 unit/kg/day plus metformin. Type of diabetes remains unknown, family history of T2DM. Patient is Hispanic.
Ordooei 2021 [77]	10 yo boy	COVID-19 with respiratory distress and diabetic ketoacidosis	Full recovery, managed post-discharge with insulin
Parappil 2022 [78]	12 yo boy	Fever, polyuria, polydipsia, diabetic ketoacidosis	Diagnosed with multisystem inflammatory syndrome and new-onset T1DM
Sarwani 2021 [79]	14 yo boy	Fever (which resolved in 1 day), abdominal pain, no respiratory symptoms	Hyperglycemia and diabetic ketoacidosis which resolved in two days; post-discharge insulin

There were fewer case studies available for adults with newly diagnosed diabetes during COVID-19. While there is a paucity of data on the course of COVID-associated diabetes after the viral infection resolves, it appears that in some cases, diabetes improves or resolves. See Table 3.

**Table 3:** Adults with COVID-19 with no history of diabetes who were diagnosed with diabetes during or soon after COVID infection. Not all studies stated the type of diabetes; when T1DM or T2DM were specified, it appears in the table. The table is organized by last name of the first author of each study [43,44,48,79-81]. yo, years old

Case Study	Patient(s)	Presentation	Case
Alshamam 2021 [80]	20 yo man	Hyperglycemia, polydipsia, polyuria; he had recently recovered from COVID-19	Full recovery from COVID-19, diagnosed with diabetes, discharged with insulin, lost to follow-up
Ghosh and Misra 2021 [81]	41 yo man	Hyperglycemia about 3 weeks after recovery from mild case of COVID-19	Treated with metformin 1 g and glimepiride 2 mg/day; after a week, glimepiride was discontinued and metformin dose reduced
Patel 2021 [43]	44 yo woman	COVID-19 with diabetic ketoacidosis; hypokalemia developed and mechanical ventilation was required	Recovered fully, requires insulin. Patient is Hispanic.
Reddy 2020 [48]	30 yo man	COVID pneumonia and diabetic ketoacidosis	Full recovery, insulin required after discharge
	60 yo man	COVID pneumonia and diabetic ketoacidosis	Full recovery, insulin required after discharge
Sarwani et al. 2021 [79]	27 yo woman	Exposed to COVID-19 but was asymptomatic; developed diabetic ketoacidosis	Recovered fully but required daily insulin. Patient had a history of gestational diabetes.
	23 yo man	COVID-19 with fatigue, weight loss, myalgia, and diabetic ketoacidosis	Recovered fully but this new-onset diabetes was thought to be an unmasking of pre-existing T1DM.
	27 yo man	Nausea, vomiting, polydipsia, polyuria, COVID-19 but no respiratory symptoms	Developed diabetic ketoacidosis which resolved in 4 days; full recovery. Insulin and metformin post-discharge
Siddiqui 2020 [44]	38 yo man	Hyperglycemia about 6 wk after recovery from COVID-19	Diagnosed and treated for T2DM. Patient had been diagnosed with prediabetes before COVID-19 infection

New-Onset Diabetes

It is not always possible to confirm new-onset virally induced diabetes in a COVID-19 patient. With limited information about the long-term course of COVID-19, data are only beginning to be collected regarding diabetes diagnosed in COVID-19 survivors who had no prior history of the disease [10,74]. The course of diabetes in COVID-19 may be transitory and self-resolving, temporary with gradual improvement, or more persistent, with risk factors for the various temporal courses unknown. Diabetes may also develop after COVID-19 resolves. In fact, people who survive COVID-19 have a 40% increased rate of developing diabetes in the next 12 months compared to those who did not have COVID-19 [82].

If diabetes is induced by COVID-19, it likely requires an interplay of multiple conditions such as stress- and/or steroid-related hyperglycemia combined with virally induced disruptions in glucose disposal and insulin secretion. Stress-related hyperglycemia is associated with insulin resistance, increased lipolysis, pronounced inflammatory response, elevated glucose production, and increased secretion of free fatty acids [83]. Damage to β-cells caused by COVID-19 infection relates to binding angiotensin-converting enzyme 2 (ACE2) receptors in the islet cells of the pancreas, increased release of glucagon from the ɑ-cells, degranulation of β-cells, and reduced secretion of insulin from the β-cells [83].

The ACE2 receptors that serve as the entry point for SARS-CoV-2 invasion are part of the renin-angiotensin-aldosterone system (RAAS), which regulates blood volume, arterial tone, blood pressure, and the balance of fluids in the body [84]. ACE2 receptors are expressed in the pancreas, allowing viral entry and damage to β-cells, resulting in hyperglycemia or other symptoms [85].

Diabetes rates increased during the pandemic; a one-year study of 47 patients under the age of 18 presenting at a clinic in Europe found that the rates of newly diagnosed diabetes were higher during the first COVID-19 wave than before the pandemic and that the cases of diabetes diagnosed during the pandemic were more severe [86]. Many children who presented with new-onset diabetes during the COVID-19 pandemic had diabetic ketoacidosis not significantly associated with patient weight [86]. The severity of COVID-19 symptoms does not appear to correlate with the likelihood of emerging diabetes; pediatric patients may have asymptomatic COVID-19 together with new-onset diabetes [72,74]. In many cases reported in the literature (see Tables 2 and 3), diabetes is not immediately diagnosed as T1DM or T2DM, implying that new-onset diabetes may be a form of diabetes that is neither unequivocally T1DM nor T2DM. New-onset diabetes that developed with or without COVID-19 was compared in 555 patients (273 of whom had COVID-19) and found that symptoms, phenotype, and C-peptide levels were similar in patients with and without COVID-19, although COVID-19 patients had greater levels of hyperglycemia [87].

Hyperglycemia in COVID-19 patients with neither history nor new diagnosis of diabetes has been reported and is associated with morbidity and mortality [8]. An extreme inflammatory response can trigger insulin-resistant hyperglycemia, which, in turn, can damage β-cells [88]. In other words, hyperglycemia apart from diabetes may trigger damage that leads to diabetes. New-onset diabetes in COVID-19 patients typically has an abrupt onset, severe but transient hyperglycemic episodes, and may include potentially life-threatening diabetic ketoacidosis; in this regard, it resembles T1DM more than T2DM [89]. For example, in a case series of six pediatric patients with the critical multisystem inflammatory syndrome in COVID-19, all developed diabetic ketoacidosis and were diagnosed with T1DM [90].

Since it is an autoimmune disorder, T1DM is often first diagnosed in childhood. In a pediatric study from Finland, new-onset T1DM and/or diabetic ketoacidosis necessitating intensive care had a pre-pandemic incidence of 2.89 per 100,000 person-years from 2016 to 2019, which increased to 9.35 per 100,000 person-years in 2020 during the pandemic [91]. The median age of these pediatric patients was 9.5 and 10.0 years, respectively, for pre-pandemic and pandemic periods. Of the 33 children in the study evaluated during the pandemic, none tested positive for COVID-19 antigens or active infection. Investigators concluded that children with T1DM are more likely to develop diabetic ketoacidosis because of lockdowns and lack of ready access to medical care rather than because of the viral infection [91]. Finland is an apt location for this study as it has the highest incidence of T1DM in the world, yet the first pandemic wave in early 2020 resulted in a relatively low case burden there. Nevertheless, Finland had pandemic lockdowns with drastically limited access to healthcare resources, which may have affected new-onset diabetes rates [91].

In a study from a single tertiary care pediatric hospital in California, retrospective data were analyzed to determine if there was an increase in newly diagnosed T1DM among children over the past six years, ending in 2021 [92]. From March 2020 to March 2021, 187 children (mean age 9.6 years, 57% female) were admitted to the hospital with new T1DM, an increase of 57% over the preceding year (n=119). Interestingly, only 2.1% (4/187) of these patients had a positive COVID-19 diagnosis upon admission [92]. A study in the United Kingdom reported a marked increase in newly diagnosed cases of T1DM in children during the pandemic and attributed this to the “slow-moving disaster” of COVID-19, but did not specify if the virus was causing new-onset T1DM or causing the circumstances that allowed T1DM in children to emerge [11].

In a study from Greece, more children were newly diagnosed with T1DM during the COVID-19 year (March 2020 to February 2021) than in the prior year (21 vs. 17 children, respectively) [93]. Diabetic ketoacidosis was likewise significantly more frequent in the COVID-19 year [93]. An Italian cross-sectional study did not find an increase in newly diagnosed diabetes patients in 2020 versus 2019 at 68 centers but did find significantly more severe diabetic ketoacidosis in 2020 (44.3%) compared to 2019 (36.1%), p=0.03 [94]. This further suggests a unique presentation of diabetes among COVID-19 patients. Rates of T1DM have been shown to increase in the aftermath of extremely stressful events, such as after the Chernobyl disaster in 1986, the Northridge earthquake in Los Angeles in 1994, and the World Trade Center disaster on September 11, 2001 [95-97]. It may be that the catastrophe of a global pandemic contributed to elevated rates of T1DM.

The Circumstances of the Pandemic

The pandemic lockdowns with quarantines, poor diets, isolation, fearfulness about the disease, social distancing, overeating, stress, alcohol consumption, and a sedentary lifestyle may have further exacerbated undiagnosed diabetes or prediabetes [98-101]. In a survey of 3,473 adults, 48% gained weight during the pandemic, and 65% of those who were overweight or very overweight before the pandemic reported weight gains during the lockdowns [102]. Adiposity is a well-known risk factor for T2DM [25]. The anxiety and even despair created by lockdowns of indeterminate length, closure of schools and businesses, financial loss, unclear and sometimes contradictory public health guidance, and fear of getting sick may have artificially increased rates of diagnosed diabetes [103]. The pandemic diverted limited healthcare resources toward the pandemic and away from other areas, limiting routine care or diagnosis of metabolic disorders. Prediabetes or symptoms suggestive of metabolic disorders may have been overlooked or testing simply deferred until after the pandemic. Telemedicine and healthcare apps were widely used during the pandemic but may have had variable utility. Telehealth was sometimes highly effective, but there were gaps in healthcare services during the lockdowns. In a case series, seven young people (ages from 9 to 23 years) who did not have COVID-19 but developed new-onset T1DM during the pandemic were successfully diagnosed using telehealth apps and continuous glucose monitoring systems [104]. These smart apps were also used to educate the families of children with new-onset T1DM during the pandemic [105,106].

Discussion

The Centers for Disease Control and Prevention already reported that new diagnoses of diabetes are 166% higher in those who had COVID-19 recently compared to those who did not. An attack by the virus on the organs, including the pancreas, may result in stress hyperglycemia, glucose derangements, and damage to the β-cells [107]. The interplay between viruses and diabetes can be complex, because viral infections may have detrimental or protective effects on the body with respect to T1DM. Virally induced diabetes is not restricted to COVID-19. For example, it is thought that enteroviruses may be able to trigger new-onset diabetes [12]. The possibility of any virus-induced diabetes poses great challenges to our healthcare system [108].

Diabetes is the most expensive chronic disease in the United States with 25% of all healthcare costs going to pay for the care of people with diabetes, estimated at well over $200 billion annually (this figure does not include lost productivity) [109,110]. Diabetes is also associated with myriad health complications such as kidney disease, cardiovascular disorders, neuropathy, and stroke [110]. For example, are recovered COVID-19 patients without hyperglycemia still elevated risk for cardiometabolic disorders after COVID-19 has passed? [111] It is disconcerting that this remains unknown. What is known is that a post-pandemic surge in the diabetic population is of great concern not just in terms of morbidity and mortality, but also with respect to our limited healthcare resources and lost productivity.

In simple terms, diabetes may be viewed as a dysfunction of the autonomic nervous system. The central and peripheral nervous systems regulate the sympathetic and parasympathetic activity in the autonomic nervous system to regulate the body’s immune responses [112]. Severe inflammation, infection, or other viral attacks can cause the sympathetic and parasympathetic systems to fall out of balance; prolonged dominance of sympathetic over parasympathetic activity could plausibly lead to poor outcomes [113]. Any acute infection, such as COVID-19, can upset the body’s homeostasis and trigger a release of cytokines and an inflammatory response [40]. When diabetes is diagnosed in the context of COVID-19, it is not always immediately apparent if this was the unmasking of a pre-existing underlying condition, the acceleration of prediabetes to diabetes by the virus, or a case of new-onset diabetes that might not have occurred otherwise. Emerging diabetes can be difficult to characterize because of its atypical presentation, uncharacteristic symptoms such as hyperglycemic episodes [63], a high rate of diabetic ketoacidosis [8], and sometimes its tendency to improve markedly or resolve entirely after recovery from COVID-19.

Our narrative review has several limitations. It is a narrative rather than a systematic review and there are few randomized clinical trials on the subject of new-onset diabetes. Long-term COVID-19 experience is lacking. Much of what we know about new-onset diabetes in the context of the pandemic is from information obtained during the pandemic when healthcare systems were overwhelmed by an influx of cases and critically ill patients to the extent that observations about diabetes, hyperglycemia, and diabetic ketoacidosis were not the subject of particular or intense study.

## Conclusions

The pandemic of COVID-19 has brought with it many forms of loss and destruction, including the potential for a new subpopulation of diabetes. Hyperglycemia and diabetic ketoacidosis often occur in the context of COVID-19 and many COVID-19 patients are diagnosed with diabetes during or soon after COVID-19 infection. It is estimated that the people who have had COVID-19, even if the case was not severe, remain at a 40% elevated risk for developing diabetes over the course of the next year. COVID-related diabetes may be pre-existing diabetes unmasked, prediabetes accelerated, or new-onset diabetes that might not have otherwise developed. Presentation is atypical with hyperglycemia and diabetic ketoacidosis and in some cases, diabetes resolves or improves greatly after recovery from COVID-19.
